# From Claims to Evidence: Re-Evaluating the Molecular Pharmacology of *Cirsium japonicum*

**DOI:** 10.3390/ijms27177717

**Published:** 2026-08-28

**Authors:** Kyung-Hee Kim, Tae-Kyung Yeo, Hwa-Seung Yoo, Byong Chul Yoo

**Affiliations:** 1 Department of Applied Chemistry, School of Science and Technology, Kookmin University, Seoul 02707, Republic of Korea; kyungheekim@kookmin.ac.kr; 2 Antibody Research Institute, Kookmin University, Seoul 02707, Republic of Korea; 3 Oculi Korean Medicine Hospital, Seoul 05655, Republic of Korea; oculi2022@naver.com; 4 East West Cancer Center, Daejeon Korean Medicine Hospital, Daejeon University, Daejeon 34520, Republic of Korea; altyhs@hanmail.net; 5 Institute of Biotechnology and Bioengineering, Sungkyunkwan University, Suwon 16419, Republic of Korea

**Keywords:** *Cirsium japonicum*, molecular pharmacology, network pharmacology, signal transduction, phytochemicals, evidence-based medicine

## Abstract

*Cirsium japonicum* Fisch. ex DC. has long been used in traditional East Asian medicine and has attracted increasing attention because of its diverse pharmacological activities, including antioxidant, anti-inflammatory, antifibrotic, metabolic regulatory, and anticancer effects. Although numerous studies have investigated its phytochemical composition and biological activities, current evidence has largely been organized according to individual compounds or disease categories, providing limited insight into the shared molecular mechanisms underlying its pleiotropic actions. In this review, we critically re-evaluate the molecular pharmacology of *C. japonicum* using an evidence-oriented framework that distinguishes experimentally supported mechanisms from pharmacological associations and emerging hypotheses. Rather than accepting changes in signaling proteins or downstream biomarkers as sufficient evidence of mechanism, we assess the strength of evidence based on reproducibility, pathway-specific interventions, genetic or pharmacological validation, and direct target engagement. Current evidence indicates that nuclear factor erythroid 2-related factor 2 (Nrf2)-mediated antioxidant responses and nuclear factor kappa B (NF-κB)-associated inflammatory responses represent the most consistently observed pathway associations, although direct molecular targets and causal pathway dependency remain insufficiently established. Evidence for AMPK/PI3K-Akt-associated metabolic regulation and TGF-β/Smad-associated antifibrotic responses is comparatively more limited. In contrast, modulation of apoptosis and autophagy is currently supported primarily by indirect or context-dependent observations. We further discuss how multiple phytochemicals converge on interconnected signaling networks regulating oxidative stress, inflammation, metabolism, tissue remodeling, and cell fate, thereby providing a systems-level explanation for the broad therapeutic potential of *C. japonicum*. Finally, we highlight the need for standardized phytochemical characterization, rigorous target validation, multi-omics integration, and artificial intelligence-assisted systems biology to establish causal molecular mechanisms and accelerate the translational development of evidence-based phytopharmaceuticals.

## 1. Introduction

Medicinal plants have historically served as an invaluable source of therapeutic agents, providing structurally diverse bioactive compounds that continue to inspire modern drug discovery [1,2,3,4,5]. Unlike single-target synthetic drugs, herbal medicines typically contain multiple phytochemicals capable of interacting with numerous molecular targets simultaneously [2,6]. This multi-component, multi-target nature has increasingly attracted attention as a potential strategy for treating complex diseases characterized by dysregulated signaling networks, including metabolic disorders, chronic inflammation, fibrosis, and cancer [7,8,9]. Consequently, understanding the molecular pharmacology underlying traditional herbal medicines has become an important research direction in both pharmacology and systems biology [3,6,10].

Among these medicinal plants, *Cirsium japonicum* Fisch. ex DC. (Asteraceae) has been extensively used in East Asian traditional medicine for centuries [11]. Traditionally known as a hemostatic herb, *C. japonicum* has also been prescribed for the treatment of hemorrhage, liver disorders, inflammatory diseases, hypertension, and various chronic conditions [11]. More recently, advances in phytochemical analysis and experimental pharmacology have substantially expanded our understanding of this species, revealing a rich repertoire of flavonoids, phenolic acids, terpenoids, polysaccharides, and other bioactive constituents with diverse pharmacological activities [11,12,13,14].

Over the past decade, an increasing number of experimental studies have demonstrated that extracts and isolated compounds from *C. japonicum* exert antioxidant, anti-inflammatory, antifibrotic, hepatoprotective, metabolic regulatory, immunomodulatory, and anticancer effects in various cellular and animal models [15,16,17,18,19]. Importantly, these biological activities are not independent phenomena but frequently converge on a relatively limited number of evolutionarily conserved signaling pathways, including nuclear factor kappa B (NF-κB), nuclear factor erythroid 2-related factor 2 (Nrf2), phosphoinositide 3-kinase/protein kinase B (PI3K/Akt), mitogen-activated protein kinases (MAPKs), AMP-activated protein kinase (AMPK), transforming growth factor-β (TGF-β), and signal transducer and activator of transcription 3 (STAT3) [7,8,9,20]. Such observations suggest that the broad therapeutic potential of *C. japonicum* may arise from coordinated modulation of interconnected molecular networks rather than isolated pharmacological actions [3,6].

Several excellent reviews have summarized the traditional uses, phytochemical composition, pharmacological activities, and, more recently, pharmacokinetic properties of *C. japonicum* [11]. While these reviews provide valuable resources for understanding the medicinal plant itself, they generally organize current knowledge according to phytochemical classes or disease categories. As a result, relatively little attention has been devoted to integrating the available evidence from the perspective of molecular targets, signaling pathways, and shared biological processes [7,8]. Such integration is increasingly important because many chronic diseases—including metabolic dysfunction-associated steatotic liver disease (MASLD), diabetes, fibrosis, immune disorders, and cancer—share common molecular mechanisms involving oxidative stress, chronic inflammation, mitochondrial dysfunction, dysregulated cell survival, and altered metabolic homeostasis [3,6,10,21,22].

The growing recognition that diverse phytochemicals can converge on common signaling hubs also highlights an important conceptual shift in herbal medicine research [7,8]. Rather than considering each biological activity as an isolated pharmacological event, it is becoming increasingly relevant to understand medicinal plants as modulators of interconnected signaling networks that collectively restore cellular homeostasis [9,10]. This network-oriented perspective not only provides mechanistic explanations for the pleiotropic effects frequently observed in herbal medicines but also offers a rational framework for identifying therapeutic opportunities across multiple disease indications [3,6].

**Literature search strategy.** A structured literature search was conducted in PubMed from database inception through 31 July 2026. Search terms included “*Cirsium japonicum*,” “*Cirsium japonicum* extract,” “linarin,” “pectolinarin,” “apigenin,” “luteolin,” and “chlorogenic acid,” used alone or in combination with terms related to molecular pharmacology and biological mechanisms, including “molecular target,” “signaling pathway,” “oxidative stress,” “Nrf2,” “NF-κB,” “MAPK,” “AMPK,” “PI3K/Akt,” “TGF-β,” “inflammation,” “fibrosis,” “metabolism,” “apoptosis,” “autophagy,” “pharmacokinetics,” “bioavailability,” and “toxicity.” The reference lists of relevant articles were also examined to identify additional primary studies. Peer-reviewed original studies were considered when they investigated *C. japonicum* extracts, fractions, or constituents identified in *C. japonicum* and reported experimentally measured pharmacological effects, signaling-associated responses, molecular biomarkers, or pharmacokinetic findings relevant to the scope of this review. Review articles were used for background information and to identify relevant primary studies but were not treated as independent mechanistic evidence. Studies were excluded from mechanistic assessment when the tested preparation, experimental model, or measured outcome was insufficiently described, or when the proposed mechanism was based exclusively on computational prediction without experimental support. Because of substantial heterogeneity in tested preparations, experimental models, doses, and outcome measures, the evidence was synthesized qualitatively rather than through quantitative meta-analysis.

**Figure 1:**
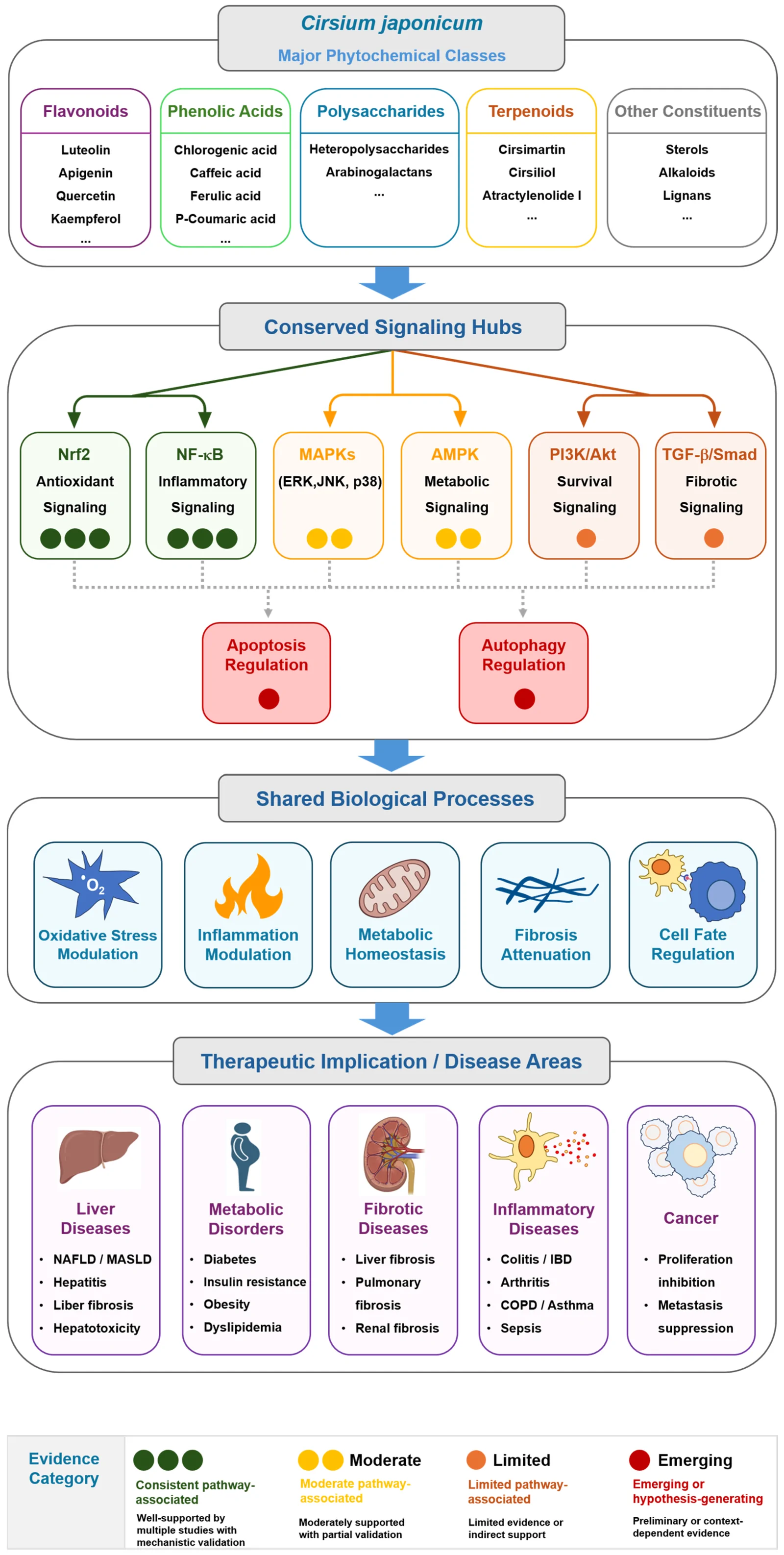
Evidence-based molecular pharmacology of *Cirsium japonicum*: linking phytochemical diversity to conserved signaling pathways and therapeutic implications. Major phytochemical classes identified in *Cirsium japonicum*, including flavonoids, phenolic acids, terpenoids, polysaccharides, and other constituents, have been associated with changes in multiple conserved signaling pathways rather than acting through a single molecular target. These signaling hubs—including Nrf2, NF-κB, MAPKs, AMPK, PI3K/Akt, TGF-β/Smad, apoptosis, and autophagy—converge on shared biological processes associated with oxidative stress, inflammation, metabolic homeostasis, fibrosis, and cell fate regulation, potentially contributing to the reported preclinical pharmacological responses in liver, metabolic, fibrotic, inflammatory diseases, and cancer. Evidence categories are indicated by color coding and reflect the consistency and depth of experimental support summarized throughout this review. These categories distinguish reproducible pathway-associated responses from limited, emerging, or context-dependent evidence and do not imply direct target engagement or established causal mechanisms. The figure was assembled and edited using Microsoft PowerPoint (Microsoft Corporation, Redmond, WA, USA), with selected graphical elements created in BioRender. Kim, K. (2026) https://BioRender.com/qaey8jz (accessed on 25 August 2026).

In this review, we critically re-evaluate the molecular pharmacology of *Cirsium japonicum* by distinguishing experimentally supported mechanisms from associations, extrapolations, and emerging hypotheses. Changes in signaling proteins or downstream biomarkers were considered valid evidence of pathway-associated pharmacological responses but were not regarded as sufficient to establish pathway dependency, direct molecular targeting, or a causal mechanism. Accordingly, we evaluated the nature and depth of the available evidence, including the chemical identity of the tested preparation, reproducibility across experimental models, pathway-specific interventions, genetic or pharmacological validation, functional rescue, and direct target engagement. Particular attention is given to widely proposed signaling mechanisms involving Nrf2, NF-κB, MAPKs, AMPK, PI3K/Akt, mTOR, TGF-β/Smad, apoptosis, and autophagy. Through this evidence-oriented framework, we aim to clarify which pharmacological claims are consistently supported at the level of pathway association, and which experimental approaches are required to establish causal molecular mechanisms and advance the translational development of *C. japonicum*. An overview of the evidence-based conceptual framework proposed in this review is presented in Figure 1.

## 2. Phytochemical Landscape as the Foundation of Molecular Pharmacology

### 2.1. Chemical Diversity of Cirsium japonicum

The therapeutic potential of medicinal plants arises not only from the presence of bioactive compounds but also from the remarkable diversity of their chemical constituents [1,3]. Unlike conventional pharmaceuticals, which are generally designed to modulate a single molecular target, medicinal plants contain numerous structurally distinct metabolites capable of interacting with multiple proteins and signaling pathways simultaneously [2,9]. Such chemical diversity provides a mechanistic basis for the pleiotropic pharmacological effects frequently observed in herbal medicines and has attracted increasing interest in the treatment of complex diseases involving dysregulated molecular networks [6,7,8].

*Cirsium japonicum* Fisch. ex DC. represents a chemically diverse medicinal plant with a broad spectrum of biologically active constituents [11]. Phytochemical investigations have identified flavonoids, phenolic acids, terpenoids, steroids, polysaccharides, and several minor metabolites from different parts of the plant [11,12,13,14]. Among these, flavonoids constitute the predominant class and include compounds such as linarin, pectolinarin, apigenin, luteolin, and acacetin, many of which have demonstrated significant biological activities in experimental models [12,13,16,17,22,23]. In addition, phenolic acids, including chlorogenic acid and caffeic acid contribute to the antioxidant properties of the plant, while polysaccharides and terpenoid constituents have been implicated in immunomodulatory and metabolic regulation [14,18,19].

Although the phytochemical composition of *C. japonicum* has been extensively characterized, simply cataloguing individual compounds provides limited insight into its pharmacological complexity [11]. Many constituents exhibit overlapping biological activities, and structurally unrelated metabolites frequently converge on common molecular targets involved in oxidative stress, inflammatory signaling, metabolic regulation, cell survival, and tissue remodeling [3,7,8]. Conversely, a single phytochemical may simultaneously regulate multiple signaling pathways depending on the biological context [9,10]. These observations indicate that the therapeutic effects of *C. japonicum* are more appropriately understood as the integrated outcome of coordinated network modulation rather than the action of isolated compounds [6,8].

Accordingly, this review does not attempt to comprehensively summarize every phytochemical identified in *C. japonicum*, as this aspect has been well documented in previous reviews [11]. Instead, we focus on representative bioactive constituents with experimentally reported pathway-associated responses and discuss how these compounds collectively modulate conserved signaling networks responsible for the diverse pharmacological activities of *C. japonicum*. This perspective provides the conceptual foundation for the following sections, in which molecular targets and signaling pathways, rather than chemical classification alone, serve as the principal framework for interpreting the biological actions of this medicinal plant. Table 1 provides a phytochemical overview of *C. japonicum* by summarizing its major chemical classes, representative constituents, and broadly reported pharmacological properties without assigning direct molecular targets or causal mechanisms.

### 2.2. Representative Phytochemicals as Functional Network Modulators

Although *C. japonicum* contains numerous phytochemicals, only a limited number have been investigated in sufficient detail to characterize their pathway-associated pharmacological responses [11,12]. These representative compounds provide a mechanistic framework for understanding how the chemical diversity of *C. japonicum* is translated into broad pharmacological activities. Rather than being associated with unique and independent mechanisms, many of these constituents have been reported to influence overlapping signaling pathways involved in cellular redox balance, inflammatory responses, energy metabolism, apoptosis, and tissue remodeling [3,7,8].

Among the flavonoids, linarin and pectolinarin have attracted considerable attention because of their diverse biological activities [12,14]. Preclinical studies have reported anti-inflammatory, antioxidant, hepatoprotective, and anticancer activities associated with changes in multiple signaling pathways, including NF-κB, MAPKs, PI3K/Akt, and Nrf2 [15,16,22,30,31,32,33]. Although the relative contribution of each pathway varies depending on the experimental model, these observations suggest that a single phytochemical may be associated with changes in multiple signaling pathways rather than a single linear signaling cascade [8,9].

Other flavonoids, particularly apigenin and luteolin, exhibit similarly pleiotropic activities [13,19]. These compounds have been reported to regulate oxidative stress, inflammatory signaling, mitochondrial function, autophagy, and programmed cell death in diverse disease models [24,25,26,34,35,36,37]. Importantly, many of these biological effects are mediated through conserved signaling hubs that are shared across different pathological conditions [3,7]. Consequently, the reported preclinical activities of individual flavonoids extend across multiple disease models and may reflect their association with cellular homeostatic processes [6,8].

Phenolic acids such as chlorogenic acid and caffeic acid have also been reported to influence cellular redox status and inflammatory signaling, thereby complementing these activities [14,19]. In parallel, polysaccharides and terpenoid constituents have been associated with immune regulation, metabolic adaptation, and tissue protection [11]. Although these classes of compounds have generally received less mechanistic attention than flavonoids, accumulating evidence suggests that they participate in the overall pharmacological profile of *C. japonicum* through complementary biological functions [3,11].

An important limitation in interpreting these pharmacological activities is the insufficient consideration of systemic exposure. In vitro modulation of signaling pathways does not necessarily indicate that the responsible phytochemicals are absorbed and reach relevant tissues at biologically effective concentrations. Many plant-derived flavonoids and phenolic compounds exhibit limited aqueous solubility, intestinal permeability, metabolic stability, or oral bioavailability, and pharmacokinetic information for *C. japonicum* extracts and their representative constituents remains incomplete. Accordingly, the observed cellular activities should not be directly extrapolated to systemic therapeutic effects without absorption, distribution, metabolism, and exposure–response data. Formulation-based approaches may help address these limitations. In particular, co-assembly of natural plant compounds into nanocomplexes through non-covalent interactions has been proposed to improve their dispersibility, stability, biocompatibility, and bioavailability [38]. However, such co-assembly strategies have not yet been systematically evaluated for *C. japonicum* constituents and should therefore be regarded as a prospective optimization approach rather than an established property of the plant preparation.

This multi-pathway pattern is also consistent with broader evidence from traditional medicines, in which multiple extracts and phytochemicals have been reported to converge on inflammatory, oxidative-stress, angiogenic, and metabolic signaling pathways across complex disease models [39].

Collectively, current evidence suggests that the pharmacological activities of *C. japonicum* cannot be attributed to a single “lead compound.” Instead, multiple phytochemicals appear to function as complementary regulators of interconnected signaling pathways, generating integrated biological responses at the cellular level [7,8,9]. This concept shifts the focus from identifying the most active constituent toward understanding how chemically distinct molecules collectively influence shared molecular networks. Such a systems-oriented perspective forms the basis for the subsequent discussion of the molecular target landscape of *C. japonicum*. This conceptual framework is illustrated in Figure 1, which summarizes how chemically diverse phytochemicals converge on conserved signaling hubs to regulate shared biological processes and ultimately contribute to the broad therapeutic potential of *C. japonicum*. The figure also highlights the relative strength of current mechanistic evidence, providing an evidence-based overview that serves as the foundation for the subsequent evaluation of individual signaling pathways.

A major limitation of the current literature is that most mechanistic studies attribute biological activities to crude extracts or incompletely characterized phytochemical mixtures [3,11]. Consequently, it often remains unclear which constituent is responsible for the reported molecular effects, whether multiple compounds act synergistically, or whether the observed signaling changes simply reflect secondary cellular responses. This limitation complicates comparisons among studies and weakens causal interpretation of many proposed molecular mechanisms. Therefore, rigorous phytochemical standardization and quantitative characterization of bioactive constituents should be considered essential prerequisites for future mechanistic investigations [6]. Such standardized experimental systems will facilitate the application of target validation approaches, including chemical proteomics, target-engagement assays, and multi-omics integration, thereby enabling more reliable identification of primary molecular targets and causal signaling mechanisms. Combined with systems biology and artificial intelligence-assisted analyses, these approaches may provide a more comprehensive understanding of how chemically diverse constituents influence complex biological networks and support the future evaluation of *C. japonicum* as a potential phytopharmaceutical resource [10]. In contrast to the chemical classification presented in Table 1, Table 2 reorganizes selected representative phytochemicals according to their reported signaling associations and related biological processes to illustrate convergence on shared pathways.

## 3. Re-Evaluating Molecular Mechanisms: From Claims to Evidence

### 3.1. Evidence Assessment Framework

Throughout this review, the strength of evidence for each proposed molecular mechanism is qualitatively evaluated according to the depth of experimental validation rather than the number of published reports alone [3,6]. Because many studies investigating medicinal plants rely primarily on changes in signaling proteins or downstream biomarkers, distinguishing causal mechanisms from pharmacological associations remains a major challenge [10]. To address this issue, the molecular mechanisms discussed throughout this review are interpreted using an evidence-oriented framework that emphasizes experimental rigor rather than descriptive observations.

The inclusion of a study in the present assessment does not imply that the study establishes a direct molecular target or a causal mechanism. Studies reporting reproducible changes in pathway components or downstream biomarkers were used to evaluate the consistency of pathway association, whereas causal evidence was assessed separately based on pathway dependency, functional intervention, and target-engagement experiments.

Evidence was evaluated qualitatively according to four predefined categories. “Consistent pathway-associated evidence” indicates that comparable pathway-related responses have been reproduced across multiple independent studies or experimental models, although direct target engagement or pathway dependency has not been established. “Moderate pathway-associated evidence” indicates that pathway-related molecular changes and associated biological effects have been reported in more than one experimental setting, but replication across independent models and pathway-specific validation remain insufficient. “Limited pathway-associated evidence” refers to findings supported by a small number of studies, predominantly downstream biomarkers, or context-dependent observations. “Emerging or hypothesis-generating evidence” refers to proposed mechanisms inferred primarily from indirect, exploratory, correlative, or computational findings without sufficient experimental validation. Causal pathway dependency was assessed separately and would require demonstration through genetic manipulation, pathway-specific pharmacological intervention, or functional rescue. Evidence of direct molecular targeting was considered separately and would additionally require biochemical or biophysical target-engagement analysis.

In this review, a pathway is considered associated with a pharmacological response when treatment is accompanied by changes in pathway-related biomarkers, such as protein expression, phosphorylation, nuclear translocation, or downstream mediator levels. These observations indicate that the pathway responds to treatment but do not establish that its modulation is necessary or sufficient for the observed phenotype. By contrast, causal pathway dependency requires pathway-specific perturbation demonstrating that inhibition or disruption of the pathway abolishes the pharmacological effect or that its restoration rescues the effect. Direct molecular targeting represents a further and distinct level of evidence requiring biochemical or biophysical demonstration of target engagement. Accordingly, pathway association, causal pathway dependency, and direct molecular targeting are treated as separate levels of mechanistic inference throughout this review.

This framework is intended to distinguish reproducible pathway-associated responses from preliminary pharmacological observations and causally validated mechanisms, thereby providing a more critical interpretation of the current literature. Rather than simply summarizing published findings, our objective is to identify which signaling pathways show consistent pathway-associated evidence, which remain speculative, and where additional mechanistic studies are required to establish causal relationships. The evidence supporting each proposed molecular mechanism is summarized in Table 3.

### 3.2. Regulation of Oxidative Stress and Cellular Redox Homeostasis

Oxidative stress is a fundamental pathological process underlying the development and progression of numerous chronic diseases, including metabolic dysfunction-associated steatotic liver disease (MASLD), cardiovascular disorders, diabetes, neurodegenerative diseases, fibrosis, and cancer [41,42]. Excessive production of reactive oxygen species (ROS) disrupts cellular redox homeostasis, resulting in oxidative damage to lipids, proteins, and nucleic acids. However, under physiological conditions, ROS also function as essential signaling molecules that regulate cell proliferation, differentiation, immune responses, and metabolic adaptation [42]. Therefore, pathological oxidative stress reflects not merely excessive ROS production but an imbalance between oxidant generation and endogenous antioxidant defense systems [41]. Beyond direct molecular injury, ROS activate multiple downstream signaling pathways involved in inflammation, apoptosis, metabolic dysfunction, and extracellular matrix remodeling. Consequently, restoration of redox homeostasis has emerged as an important therapeutic strategy for preventing chronic disease progression [43]. As summarized in Table 3, the following sections critically evaluate the strength of evidence supporting each proposed molecular mechanism rather than simply cataloguing reported signaling changes.

Accumulating evidence indicates that *C. japonicum* attenuates oxidative stress through multiple complementary mechanisms [11]. In addition to directly scavenging reactive oxygen species, extracts and isolated phytochemicals from *C. japonicum* enhance endogenous antioxidant defense systems by regulating redox-sensitive signaling pathways and antioxidant enzymes [12,16,17]. These observations suggest that the antioxidant activity of *C. japonicum* extends beyond simple free radical neutralization and involves active modulation of intracellular redox homeostasis rather than passive antioxidant effects [3].

Among the redox-sensitive signaling pathways, the nuclear factor erythroid 2-related factor 2 (Nrf2) pathway represents one of the principal cellular defense mechanisms against oxidative stress [43,44]. Under physiological conditions, Nrf2 activity is tightly controlled by Kelch-like ECH-associated protein 1 (Keap1), whereas oxidative or electrophilic stimuli promote Nrf2 stabilization and nuclear translocation, leading to transcriptional activation of numerous cytoprotective genes, including HO-1, NQO1, GCLC, GCLM, SOD, GPXs, and enzymes involved in glutathione metabolism [44,45]. Activation of this pathway enhances cellular resistance to oxidative injury while simultaneously attenuating inflammatory signaling, preserving mitochondrial function, and maintaining metabolic homeostasis [43].

Several studies have reported that extracts or major phytochemicals of *C. japonicum*, including flavonoids (e.g., linarin and pectolinarin) and phenolic acids (e.g., chlorogenic acid), activate Nrf2-dependent antioxidant responses in various experimental models [16,17,19,46,47,48,49]. Although the experimental models differ considerably, activation of the Nrf2/ARE axis represents one of the most consistently reproduced molecular responses observed following treatment with *C. japonicum* extracts or major flavonoids [11]. Enhanced expression of HO-1, NQO1, SOD, and related antioxidant enzymes has been associated with reduced intracellular ROS accumulation, preservation of mitochondrial integrity, and improved cellular survival under oxidative stress conditions [16,19,22,50]. Although the precise upstream mechanisms responsible for Nrf2 activation remain incompletely understood, these findings consistently support the role of *C. japonicum* as a regulator of endogenous antioxidant defense rather than merely a source of exogenous radical scavengers [3].

Importantly, regulation of oxidative stress also provides a mechanistic link between multiple downstream pharmacological activities attributed to *C. japonicum*. Suppression of ROS accumulation limits activation of redox-sensitive inflammatory pathways such as NF-κB and MAPKs, reduces cytokine production, mitigates fibrogenic signaling, and contributes to the maintenance of metabolic homeostasis [11,43]. Therefore, modulation of cellular redox balance should be regarded not as an isolated pharmacological effect but as an upstream molecular event that integrates many of the subsequent biological actions discussed in this review.

Current evidence indicates that Nrf2-associated antioxidant signaling represents one of the most consistently reproduced pathway-associated responses following *C. japonicum* treatment. However, the strength of evidence varies substantially among studies. Most reports demonstrate increased Nrf2 expression or nuclear localization together with upregulation of downstream antioxidant enzymes such as HO-1 and NQO1. These changes are interpreted here as downstream indicators of an Nrf2-associated antioxidant response and do not demonstrate direct molecular binding to Nrf2, Keap1, or other pathway components. Moreover, Nrf2 activation is frequently inferred from increased expression of downstream antioxidant enzymes without excluding the contribution of alternative upstream pathways that may independently regulate these targets. Relatively few studies employ pathway-specific approaches, including Nrf2 knockdown, pharmacological inhibition, genetic loss-of-function models, or direct analyses of Keap1–Nrf2 interactions, to establish causality. Consequently, although the available evidence strongly supports an association between *C. japonicum* treatment and activation of the Nrf2 antioxidant program, the precise upstream molecular events responsible for this response remain incompletely defined. Future studies incorporating pathway-specific validation and direct target-engagement analyses will be essential to establish the causal molecular mechanisms underlying Nrf2 activation by *C. japonicum*.

### 3.3. Modulation of Inflammatory Signaling Networks

Persistent inflammation is a hallmark of numerous chronic diseases and is closely intertwined with oxidative stress, metabolic dysfunction, fibrosis, and tumor progression [51,52]. Unlike acute inflammation, which is essential for host defense and tissue repair, chronic low-grade inflammation promotes sustained cytokine production, immune cell activation, and progressive tissue injury. These pathological responses are primarily orchestrated through interconnected signaling pathways, among which nuclear factor-κB (NF-κB) and mitogen-activated protein kinases (MAPKs) serve as central regulatory hubs controlling inflammatory gene expression [53,54].

NF-κB functions as a master transcriptional regulator of inflammatory responses by controlling the expression of numerous pro-inflammatory mediators, including tumor necrosis factor-α (TNF-α), interleukin (IL)-1β, IL-6, inducible nitric oxide synthase (iNOS), and cyclooxygenase-2 (COX-2) [53]. Under inflammatory conditions, activation of the IκB kinase (IKK) complex induces phosphorylation and degradation of IκB proteins, allowing NF-κB to translocate into the nucleus and initiate inflammatory gene transcription. In parallel, MAPK family members, including extracellular signal-regulated kinase (ERK), c-Jun N-terminal kinase (JNK), and p38 MAPK, regulate inflammatory gene expression through phosphorylation of multiple downstream transcription factors [54]. Extensive crosstalk between NF-κB and MAPK pathways amplifies inflammatory signaling and contributes to the persistence of chronic inflammatory responses.

Accumulating experimental evidence indicates that *C. japonicum* suppresses inflammatory responses in diverse cellular and animal models [11]. Both crude extracts and purified phytochemicals reduce the expression of pro-inflammatory cytokines and inflammatory enzymes while attenuating activation of NF-κB and MAPK signaling pathways [15,17,27,28,30,31,32]. These anti-inflammatory effects have been observed in macrophages, hepatocytes, endothelial cells, and multiple disease models, suggesting that inflammatory pathway modulation represents a conserved pharmacological property of *C. japonicum* rather than a tissue-specific phenomenon [11].

Among the bioactive constituents, flavonoids have been most extensively investigated for their anti-inflammatory mechanisms. Representative compounds such as linarin, pectolinarin, apigenin, and luteolin have been reported to inhibit phosphorylation of IKK and IκB, suppress nuclear translocation of NF-κB, and reduce activation of ERK, JNK, and p38 MAPK [15,27,28,30,31,32]. Although the relative contribution of individual compounds differs among experimental systems, these observations indicate that multiple phytochemicals converge on common inflammatory signaling hubs. Such convergence may explain why chemically distinct constituents often produce comparable anti-inflammatory outcomes despite differences in molecular structure, suggesting that regulation of signaling networks rather than single molecular targets may underlie the anti-inflammatory activity of *C. japonicum* [10].

Importantly, suppression of inflammatory signaling by *C. japonicum* should not be interpreted as an isolated pharmacological effect. Chronic inflammation is mechanistically linked to oxidative stress, mitochondrial dysfunction, metabolic reprogramming, extracellular matrix remodeling, and dysregulated cell survival. Consequently, inhibition of NF-κB and MAPK signaling is likely to influence a broad range of downstream biological processes beyond cytokine production alone. Moreover, reciprocal interactions between the Nrf2 antioxidant pathway and NF-κB signaling suggest that restoration of redox homeostasis may contribute indirectly to the anti-inflammatory effects of *C. japonicum*, further emphasizing the integrated nature of these signaling networks [44]. This interconnected signaling landscape provides a mechanistic basis for understanding the diverse therapeutic effects of *C. japonicum* across seemingly unrelated disease conditions and establishes a conceptual bridge to the metabolic and fibrotic signaling pathways discussed in the following sections.

**Evidence assessment.** Current evidence consistently supports the anti-inflammatory activity of *C. japonicum*, particularly through suppression of NF-κB and MAPK signaling. However, most studies rely on reduced phosphorylation of pathway components, altered nuclear translocation, and decreased expression of inflammatory mediators as surrogate markers of pathway-associated inhibition. These downstream responses do not constitute target engagement or demonstrate direct molecular binding to NF-κB pathway components. Furthermore, inhibition of NF-κB and MAPK signaling is often interpreted as the primary mechanism without excluding the possibility that these changes occur secondary to upstream modulation of oxidative stress or other signaling pathways. Direct evidence demonstrating that NF-κB or MAPK signaling is required for the observed biological effects, including pathway-specific genetic manipulation, selective pharmacological inhibition, or functional rescue experiments, remains limited. Consequently, although suppression of NF-κB and MAPK signaling represents one of the most consistently reproduced molecular responses following *C. japonicum* treatment, the available evidence currently supports a robust pharmacological association rather than a definitively established causal mechanism. Future studies employing pathway-specific validation and target-engagement approaches will be necessary to define the precise molecular events responsible for these anti-inflammatory effects.

### 3.4. Regulation of Metabolic Homeostasis

Maintenance of metabolic homeostasis requires the coordinated regulation of nutrient sensing, energy production, and biosynthetic pathways. Disruption of these regulatory systems contributes to the pathogenesis of numerous metabolic disorders, including obesity, insulin resistance, type 2 diabetes, and metabolic dysfunction-associated steatotic liver disease (MASLD) [55,56]. At the cellular level, metabolic adaptation is orchestrated by an integrated signaling network involving AMP-activated protein kinase (AMPK), phosphoinositide 3-kinase (PI3K)/Akt, mechanistic target of rapamycin (mTOR), and their downstream effectors. Rather than functioning independently, these pathways continuously integrate nutrient availability, energy status, and growth signals to maintain metabolic homeostasis [55,57].

Among these metabolic regulators, AMPK serves as the principal cellular energy sensor coordinating the transition between anabolic and catabolic metabolism [55]. Activation of AMPK promotes fatty acid oxidation, glucose uptake, mitochondrial biogenesis, and autophagy while suppressing ATP-consuming anabolic processes such as lipid, protein, and cholesterol synthesis [58]. Through these coordinated actions, AMPK restores cellular energy balance and improves metabolic flexibility. Consequently, pharmacological activation of AMPK has emerged as an attractive therapeutic strategy for metabolic diseases characterized by lipid accumulation, mitochondrial dysfunction, and impaired energy metabolism [58].

Growing evidence suggests that treatment with *C. japonicum* extracts or representative phytochemicals is consistently associated with activation of AMPK-related signaling pathways and improvements in metabolic parameters across diverse experimental models [11]. Experimental studies have demonstrated that plant extracts and representative phytochemicals improve lipid metabolism, reduce hepatic lipid accumulation, enhance glucose utilization, and attenuate metabolic dysfunction in cellular and animal models [24,25,29,33]. These metabolic improvements are frequently accompanied by increased AMPK phosphorylation, suppression of lipogenic regulators such as sterol regulatory element-binding protein-1c (SREBP-1c), and preservation of mitochondrial function. However, whether AMPK activation represents a primary molecular target of *C. japonicum* or a secondary consequence of improved cellular homeostasis remains unresolved.

In addition to AMPK, components of the PI3K/Akt signaling pathway have also been implicated in the metabolic actions of *C. japonicum*. PI3K/Akt signaling plays pivotal roles in insulin signaling, glucose metabolism, cell survival, and protein synthesis, whereas extensive crosstalk among PI3K/Akt, AMPK, and mTOR integrates nutrient sensing with cellular growth and metabolic adaptation [57,59]. The ability of *C. japonicum* phytochemicals to influence multiple components of this interconnected network suggests that their metabolic actions are unlikely to arise from modulation of a single signaling pathway. Instead, they appear to reflect coordinated regulation of metabolic signaling networks, consistent with the systems-level pharmacological effects commonly observed for medicinal plants [10].

Importantly, metabolic regulation cannot be considered independently of oxidative stress and inflammation. Persistent oxidative injury and chronic inflammatory signaling profoundly impair mitochondrial function, lipid metabolism, and insulin responsiveness, thereby establishing a vicious cycle that accelerates metabolic disease progression. Conversely, restoration of metabolic homeostasis attenuates oxidative stress, suppresses inflammatory signaling, and limits subsequent fibrogenic responses. These reciprocal interactions position metabolic signaling as a central node linking the antioxidant and anti-inflammatory properties of *C. japonicum* to its protective effects in chronic metabolic diseases.

**Evidence assessment.** Compared with antioxidant (Nrf2) and anti-inflammatory (NF-κB/MAPK) signaling, mechanistic evidence supporting metabolic regulation through AMPK and PI3K/Akt remains less mature. Most available studies report increased phosphorylation of signaling proteins together with improvements in metabolic phenotypes, but these observations do not establish whether pathway activation is causally responsible for the observed biological effects. Furthermore, pathway-specific inhibition, genetic manipulation, and functional rescue experiments have rarely been performed. Consequently, although activation of AMPK-associated signaling is consistently correlated with the metabolic benefits of *C. japonicum*, current evidence supports moderate pathway-associated evidence rather than a definitive molecular mechanism. Future studies combining pathway-specific validation with direct target-identification approaches will be necessary to determine whether AMPK and related metabolic regulators function as primary molecular targets or downstream mediators of the metabolic effects of *C. japonicum*.

### 3.5. Regulation of Fibrosis and Tissue Remodeling

Fibrosis represents a common pathological outcome of persistent tissue injury and chronic inflammation, regardless of the affected organ [60,61]. Progressive accumulation of extracellular matrix (ECM) components disrupts normal tissue architecture, ultimately leading to irreversible organ dysfunction. Liver, pulmonary, renal, and cardiac fibrosis share highly conserved molecular mechanisms despite differences in etiology, highlighting fibrosis as a generalized pathological process rather than an organ-specific disease [60].

Transforming growth factor-β (TGF-β) is widely recognized as the principal profibrotic cytokine responsible for initiating and sustaining fibrogenesis [62]. Activation of the TGF-β/Smad signaling pathway promotes fibroblast-to-myofibroblast differentiation, stimulates excessive collagen synthesis, and suppresses extracellular matrix degradation. In addition to canonical Smad signaling, TGF-β extensively interacts with MAPKs, PI3K/Akt, NF-κB, and redox-sensitive signaling pathways, forming an integrated regulatory network that coordinates tissue remodeling [62,63,64]. Consequently, effective antifibrotic intervention is unlikely to result from inhibition of a single signaling pathway but instead requires coordinated modulation of multiple interconnected molecular processes.

Emerging evidence suggests that treatment with *C. japonicum* attenuates pathological tissue remodeling in several experimental models [11]. Experimental studies have demonstrated reduced scar formation, altered collagen organization, decreased expression of fibrogenic markers such as α-SMA, and suppression of scar-associated YAP signaling following treatment with representative phytochemicals of *C. japonicum* [40]. Although mechanistic investigations remain relatively limited compared with studies of antioxidant and anti-inflammatory signaling, the available evidence consistently supports the antifibrotic potential of *C. japonicum*.

Importantly, the antifibrotic activity of *C. japonicum* is unlikely to result from direct inhibition of TGF-β signaling alone. Reduction in oxidative stress limits activation of redox-sensitive profibrotic pathways, whereas suppression of chronic inflammation decreases the sustained production of cytokines and growth factors that promote extracellular matrix remodeling. Simultaneously, restoration of metabolic homeostasis alleviates mitochondrial dysfunction and lipotoxicity, both of which contribute to fibrogenesis in chronic metabolic disorders such as MASLD. These reciprocal interactions suggest that the antifibrotic effects of *C. japonicum* arise primarily through coordinated regulation of upstream pathological processes rather than selective blockade of downstream fibrosis signaling.

From the perspective of molecular pharmacology, fibrosis therefore represents an integrated biological consequence of multiple upstream signaling events rather than an isolated therapeutic target. This concept provides a mechanistic explanation for why phytochemicals capable of simultaneously modulating oxidative stress, inflammation, and metabolism frequently exert broad protective effects on tissue remodeling. Consistent with a systems pharmacology perspective, the antifibrotic activity of *C. japonicum* is therefore best interpreted as the cumulative outcome of network-level regulation rather than selective inhibition of an individual profibrotic pathway [10].

**Evidence assessment.** Compared with antioxidant and anti-inflammatory signaling, mechanistic evidence supporting antifibrotic activity remains relatively limited. Most studies report reduced collagen deposition, decreased α-SMA expression, altered extracellular matrix remodeling, and histological improvement. However, these observations do not establish whether modulation of TGF-β signaling is the primary driver of the antifibrotic response or a downstream consequence of reduced oxidative stress, inflammation, or metabolic dysfunction. Pathway-specific inhibition, genetic manipulation, and functional rescue experiments remain scarce. Consequently, although current evidence consistently supports the antifibrotic potential of *C. japonicum*, the underlying molecular mechanisms should presently be regarded as limited mechanistic evidence rather than definitively established causal pathways. Future studies integrating pathway-specific validation with target-identification approaches will be required to determine how *C. japonicum* regulates fibrogenesis at the molecular level.

### 3.6. Regulation of Cell Fate: Apoptosis, Autophagy, and Survival Signaling

The maintenance of tissue homeostasis ultimately depends on the precise regulation of cell fate. Under physiological conditions, cellular survival, apoptosis, autophagy, and proliferation are tightly coordinated to preserve tissue integrity and function [65,66]. However, persistent oxidative stress, chronic inflammation, and metabolic dysregulation disrupt this balance, leading to excessive cell death, impaired tissue repair, or uncontrolled cellular proliferation. Consequently, dysregulation of cell fate has emerged as a common pathological feature of numerous chronic diseases, including metabolic disorders, fibrosis, neurodegenerative diseases, and cancer [65,67].

Apoptosis is one of the most extensively studied forms of programmed cell death and is primarily regulated through the intrinsic mitochondrial pathway and the extrinsic death receptor pathway [67]. Cellular stress activates a complex signaling network involving Bcl-2 family proteins, cytochrome *c* release, caspase activation, and multiple upstream kinases, including PI3K/Akt, MAPKs, and p53. Depending on the pathological context, either excessive apoptosis or resistance to apoptosis contributes to disease progression. Therefore, restoration of appropriate apoptotic regulation, rather than simple inhibition or activation of cell death, represents an important therapeutic objective.

Autophagy constitutes another essential adaptive mechanism that maintains cellular quality control through the lysosomal degradation of damaged proteins and dysfunctional organelles [68]. Beyond its role in nutrient recycling, autophagy contributes to mitochondrial homeostasis, redox regulation, immune responses, and metabolic adaptation. Increasing evidence demonstrates extensive crosstalk among autophagy, apoptosis, AMPK, mTOR, and oxidative stress signaling, indicating that these pathways collectively determine cellular responses to physiological and pathological stress [65,68].

Experimental studies indicate that *C. japonicum* influences multiple components of these interconnected cell fate pathways [11]. Extracts and representative phytochemicals have been reported to attenuate oxidative stress-induced cellular injury, preserve mitochondrial function, and modulate apoptosis- and autophagy-associated signaling pathways [22,23]. In several disease models, these effects are accompanied by activation of cytoprotective signaling pathways and suppression of excessive inflammatory responses, thereby promoting cellular survival under pathological conditions. Conversely, studies using cancer models have demonstrated that *C. japonicum* phytochemicals can induce apoptosis, inhibit tumor cell proliferation, and alter survival signaling, indicating that their biological actions are highly dependent on cellular context.

These context-dependent effects illustrate an important principle of molecular pharmacology. Rather than functioning as nonspecific inhibitors or activators of cell death, the bioactive constituents of *C. japonicum* appear to restore disrupted cellular homeostasis by selectively modulating signaling networks according to the underlying pathological state. In non-malignant tissues, this regulation favors cytoprotection and tissue preservation, whereas in malignant cells it promotes apoptosis and limits uncontrolled proliferation. This bidirectional regulation is consistent with the network-based pharmacological actions proposed throughout this review and further supports the concept that the therapeutic versatility of *C. japonicum* arises from coordinated modulation of conserved signaling pathways rather than from single-target mechanisms [10].

**Evidence assessment.** Current evidence suggests that *C. japonicum* modulates apoptosis- and autophagy-associated signaling in a context-dependent manner. However, most studies rely on changes in apoptosis-related proteins (e.g., Bcl-2, Bax, or caspases) or autophagy markers (e.g., LC3-II or p62) without determining whether these changes reflect primary pathway regulation or secondary responses to reduced oxidative stress, inflammation, or metabolic dysfunction. Moreover, relatively few investigations directly assess autophagic flux or employ pathway-specific genetic or pharmacological interventions to establish causality. Consequently, the available evidence supports context-dependent pharmacological associations rather than definitively established molecular mechanisms. Future studies integrating functional analyses of autophagic flux, pathway-specific validation, and target-identification approaches will be essential to clarify how *C. japonicum* regulates cell fate under different pathological conditions.

## 4. Translational Implications of Multi-Target Pharmacology

The molecular targets discussed in the preceding sections should not be regarded as independent signaling pathways but rather as components of an integrated regulatory network that governs cellular homeostasis [7,8]. Oxidative stress, inflammatory responses, metabolic adaptation, tissue remodeling, and cell fate are dynamically interconnected through extensive signaling crosstalk. Consequently, modulation of one pathway frequently influences multiple downstream biological processes. This systems-level organization provides a conceptual framework for interpreting the broad pharmacological activities reported for *C. japonicum* across diverse preclinical disease models and highlights why reductionist single-pathway models are often insufficient to explain the pharmacological actions of complex herbal medicines [10].

Unlike conventional single-target agents, herbal preparations contain multiple phytochemicals that may simultaneously influence numerous signaling pathways and biological processes [3,7]. Such polypharmacology has traditionally been regarded as a challenge for mechanistic investigation because it complicates identification of a single principal active constituent. However, this characteristic has been proposed as a potential advantage in the investigation of chronic diseases in which multiple dysregulated signaling pathways coexist and reinforce one another, although its therapeutic relevance has not been clinically established. In this context, the pharmacological activity of *C. japonicum* is better interpreted as coordinated modulation of biological networks rather than as the additive effects of individual compounds [10].

The systems-level and reductionist approaches should not be regarded as mutually exclusive. Network and multi-omics analyses can identify candidate components, pathways, and interactions within a complex pharmacological response, whereas reductionist interventions can determine whether individual nodes are necessary or sufficient for particular components of that response. Thus, single-gene knockout or pathway-specific inhibition is not intended to reduce the overall activity of *C. japonicum* to a single target, but to test causal relationships within the broader network. Conversely, the absence of a complete loss of activity following disruption of one node would not necessarily exclude a multi-component mechanism, because compensatory or parallel pathways may remain active. Validation of network-level effects will require fractionation and reconstitution studies, component-combination experiments, and simultaneous or sequential perturbation of multiple candidate pathways. Intermediate in vivo models, including simple organisms, may provide useful platforms for initial assessment of multi-component biological effects before validation in mammalian disease models. Importantly, coordinated responses to multiple constituents should not be described as pharmacological synergy unless synergy has been demonstrated using appropriately designed component controls, dose–response matrices, and quantitative interaction analyses.

This concept is particularly relevant for chronic disorders such as MASLD, diabetes, fibrosis, cardiovascular disease, neurodegenerative diseases, and cancer. Although these diseases differ clinically, they share common pathological mechanisms, including oxidative stress, chronic inflammation, mitochondrial dysfunction, impaired metabolic homeostasis, and dysregulated cell fate. The mechanistic evidence summarized in this review suggests that the most consistent pathway-associated evidence currently concerns Nrf2-mediated antioxidant responses and NF-κB-associated inflammatory responses. MAPK- and AMPK-associated responses are moderately supported, whereas evidence concerning PI3K/Akt-mediated metabolic regulation and fibrotic remodeling remains limited. Evidence for apoptosis and autophagy remains emerging and context dependent. Accordingly, the available preclinical evidence supports viewing *C. japonicum* as a source of compounds associated with multiple signaling pathways, while pathway dependency, direct molecular targets, and clinical relevance remain to be established.

Recent advances in systems biology provide useful approaches for investigating complex pharmacological responses [69]. Multi-omics technologies—including transcriptomics, proteomics, metabolomics, and lipidomics—can provide comprehensive profiles of molecular changes following treatment with herbal medicines. Integration of these datasets with systems pharmacology, network analysis, and computational pathway modeling may prioritize candidate signaling hubs, molecular interactions, and pharmacodynamic biomarkers for subsequent investigation [10]. However, database-derived compound–target associations, network centrality, enrichment analysis, and molecular docking remain hypothesis-generating approaches. These methods cannot independently demonstrate physical binding, target engagement, pathway dependency, or causal therapeutic mechanisms. Accordingly, computationally identified molecules should be described as predicted targets or candidate network hubs until they are validated using biochemical binding assays, target-engagement approaches, genetic or pharmacological perturbation, and functional rescue experiments.

Artificial intelligence and machine learning further provide opportunities to integrate high-dimensional biological datasets into predictive models of therapeutic response [70]. Rather than evaluating individual compounds or isolated signaling pathways, these computational approaches can analyze complex relationships among phytochemicals, molecular targets, biological pathways, and disease phenotypes. When combined with experimentally validated molecular data, such integrative strategies may facilitate the prioritization of candidate mechanisms and biomarkers for further preclinical and clinical investigation.

Despite these advances, several important challenges remain. Most mechanistic studies have relied on crude extracts or individual phytochemicals evaluated in isolated experimental systems, making direct comparison among studies difficult. Furthermore, the relative contributions of individual constituents, synergistic or antagonistic interactions, pharmacokinetic behavior, tissue distribution, bioavailability, and long-term safety remain insufficiently characterized. Although systems pharmacology provides a valuable framework for generating mechanistic hypotheses, computational predictions must ultimately be supported by rigorous experimental validation, including pathway-specific intervention, target-engagement analyses, and functional studies. Addressing these limitations will require standardized phytochemical characterization together with systematic integration of molecular, pharmacological, and clinical evidence.

Collectively, current evidence supports a transition from descriptive phytochemical research toward mechanism-driven molecular pharmacology. Rather than acting through a single dominant molecular target, *C. japonicum* appears to regulate a network of conserved signaling pathways that collectively maintain cellular homeostasis. This network-based perspective provides a framework for interpreting its reported biological activities and for designing future studies that evaluate its pharmacological and translational potential. The major translational challenges and future research priorities are summarized in Table 4.

## 5. Conclusions and Future Perspectives

*Cirsium japonicum* has long been recognized as a valuable medicinal plant in traditional East Asian medicine, and growing experimental evidence has substantially expanded our understanding of its biological activities [11]. Although previous studies have documented diverse pharmacological effects, including antioxidant, anti-inflammatory, hepatoprotective, antifibrotic, metabolic regulatory, and anticancer activities, these findings have often been interpreted as independent observations associated with individual disease models or isolated phytochemicals. While this body of work has generated extensive descriptive knowledge, it has provided only limited insight into the conserved molecular mechanisms that may unify these diverse biological effects [3].

In this review, we have re-examined *C. japonicum* from the perspective of molecular pharmacology. Rather than organizing current knowledge according to phytochemical classes or disease categories, we synthesized the available evidence around conserved signaling pathways that regulate oxidative stress, inflammation, metabolism, tissue remodeling, and cell fate. This evidence-based framework suggests that chemically diverse phytochemicals frequently converge on a relatively limited number of signaling hubs—including Nrf2, NF-κB, MAPKs, AMPK, PI3K/Akt, mTOR, and TGF-β—to collectively maintain cellular homeostasis. Importantly, the strength of pathway-associated evidence differs substantially among these pathways, emphasizing the need to distinguish reproducible signaling associations from causal mechanisms that remain unvalidated.

This network-oriented perspective provides a conceptual framework for interpreting the diverse preclinical activities reported for *C. japonicum*. Rather than functioning through a single dominant molecular target, *C. japonicum* appears to modulate interconnected signaling networks that influence multiple pathological processes simultaneously. Such systems-level associations may be relevant to the investigation of chronic diseases characterized by overlapping pathogenic mechanisms, including oxidative stress, inflammation, metabolic dysfunction, fibrosis, mitochondrial impairment, and dysregulated cell survival [7,10].

Despite the reported pharmacological activities, the current evidence remains predominantly preclinical. Direct molecular targets of many phytochemicals have yet to be conclusively identified, and the relative contributions of individual constituents within complex extracts remain unclear. Moreover, pharmacokinetic profiles, systemic exposure, tissue distribution, oral bioavailability, dose–response relationships, long-term safety, and potential herb–drug interactions remain insufficiently characterized. Differences in extraction methods, chemical composition, and dosing further hinder comparisons among studies, and controlled clinical investigations are lacking. Therefore, the available findings should be interpreted as evidence of pharmacological potential rather than established therapeutic or clinical efficacy, and no clinical recommendation regarding the use of *C. japonicum* can currently be made.

Future investigations should therefore move beyond documenting changes in signaling-associated biomarkers by combining systems-level identification of candidate network interactions with targeted validation using genetic manipulation, pathway-specific intervention, direct target-engagement analyses, and functional rescue experiments. Such reductionist approaches should be used to define the contribution of individual network nodes, while multi-component reconstitution and combinatorial perturbation studies are needed to determine how these nodes collectively contribute to the overall pharmacological response. Integrating these approaches with systems pharmacology will be essential for distinguishing pharmacological associations from genuine mechanisms of action and for accelerating the translation of *C. japonicum* from experimental research to evidence-based phytopharmaceutical development.

Collectively, the current evidence supports a conceptual transition from descriptive phytochemistry toward mechanism-based molecular pharmacology. By integrating phytochemicals, molecular targets, signaling pathways, and biological processes into a unified systems-level framework, this review provides a mechanistic interpretation of the pharmacological actions of *C. japonicum*. More importantly, it emphasizes that the future advancement of *C. japonicum* research will depend not on identifying additional biological activities but on establishing causal molecular mechanisms through rigorous experimental validation. Such a transition will be essential for realizing the full translational potential of *C. japonicum* in precision phytomedicine.

## Figures and Tables

**Table 1:** Phytochemical Classes and Representative Constituents Identified in *Cirsium japonicum*.

Compound/Class	Representative Compounds	Chemical Class	Representative Pharmacological Properties	Key Ref.
**Flavonoids**	Linarin	Flavone glycoside	Antioxidant, anti-inflammatory, hepatoprotective	[16,22]
	Pectolinarin	Flavone glycoside	Anti-inflammatory, anticancer	[12,14]
	Apigenin	Flavone	Metabolic regulation, antifibrotic activity	[24,25,26]
	Luteolin	Flavone	Anti-inflammatory, immunomodulatory	[27,28]
**Phenolic acids**	Chlorogenic acid	Phenolic acid	Antioxidant, glucose/lipid metabolism	[19,29]
	Caffeic acid	Phenolic acid	Anti-inflammatory, ROS scavenging	[11]
**Polysaccharides**	CJ polysaccharides	Polysaccharide	Immunomodulatory, antioxidant	[11]
**Others**	Terpenoids, sterols, volatile compounds	Miscellaneous	Emerging pharmacological activities	[11]

**Table 2:** Reported Signaling Associations of Representative *Cirsium japonicum* Phytochemicals.

Representative Phytochemical	Reported Signaling Hub	Major Signaling Pathways	Biological Processes Affected	Potential Pharmacological Relevance	Key Ref.
Linarin	Nrf2, NF-κB	Nrf2/HO-1, NF-κB	Oxidative stress, inflammation	Liver protection, chronic inflammation	[16,22,30]
Pectolinarin	NF-κB, YAP, En1	NF-κB; YAP/En1-associated remodeling	Inflammation, tissue remodeling	Anti-inflammatory activity, wound healing	[12,14,40]
Apigenin	AMPK, PI3K/Akt	AMPK, Akt	Metabolism, apoptosis	Metabolic diseases, cancer	[24,25,34,35]
Luteolin	NF-κB, MAPKs	ERK/JNK/p38	Inflammation, immune-cell activation	Inflammatory and neuroinflammatory disorders	[27,28]
Chlorogenic acid	AMPK, Nrf2	AMPK, Nrf2	Lipid metabolism, oxidative stress	Metabolic disorders	[19,29]

Note: The listed signaling molecules and pathways represent reported pathway-associated responses and should not be interpreted as directly bound or causally validated molecular targets.

**Table 3:** Evidence Assessment of Pathway-Associated Pharmacological Responses Reported for *Cirsium japonicum*.

Pathway or Biological Process Evaluated	Representative Evidence	Key Evidence Still Lacking	Evidence Interpretation	Key Ref.
Nrf2 antioxidant signaling	Increased Nrf2 nuclear localization; upregulation of HO-1 and NQO1; reduced intracellular ROS	Genetic validation (Nrf2 knockdown/knockout); direct target engagement	**Consistent pathway-associated evidence**	[16,19,22]
NF-κB inflammatory signaling	Reduced NF-κB nuclear translocation; decreased IκB degradation; reduced TNF-α, IL-1β, IL-6	Direct target engagement; pathway-specific rescue experiments	**Consistent pathway-associated evidence**	[15,30,32]
MAPK signaling	Reduced ERK, JNK, and p38 phosphorylation	Pharmacological inhibition or genetic validation demonstrating causal involvement	**Moderate pathway-associated evidence**	[15,27]
AMPK metabolic regulation	Increased AMPK phosphorylation; reduced ACC phosphorylation; improved metabolic phenotypes	Pharmacological inhibition (e.g., Compound C); genetic validation; upstream target identification	**Moderate pathway-associated evidence**	[24,29]
PI3K/Akt signaling	Altered Akt phosphorylation associated with improved metabolic or survival responses	Functional validation; pathway-specific intervention; direct target identification	**Limited pathway-associated evidence**	[33,35]
Fibrotic remodeling signaling	Reduced α-SMA expression, altered collagen organization, decreased YAP and En1 expression	Direct target validation; pathway-specific inhibition; genetic validation	**Limited pathway-associated evidence**	[26,40]
Autophagy regulation	Altered LC3-II, Beclin-1, and autophagy-associated proteins	Autophagic flux analysis; genetic manipulation (ATG genes); mechanistic validation	**Emerging or hypothesis-generating evidence**	[22,33]
Apoptosis regulation	Altered Bcl-2/Bax balance, caspase activation, and apoptosis-associated proteins in a context-dependent manner	Functional validation; pathway-specific intervention; direct target identification	**Emerging or hypothesis-generating evidence**	[22,23]

Note: Evidence categories reflect the consistency and depth of pathway-associated experimental observations and do not imply direct molecular binding or established causal pathway dependency. None of the pathways evaluated in the current literature fully meets the criteria for causal pathway dependency, and direct molecular targets remain insufficiently established. Abbreviations: ACC, acetyl-CoA carboxylase; α-SMA, alpha-smooth muscle actin; ERK, extracellular signal-regulated kinase; HO-1, heme oxygenase-1; IL, interleukin; JNK, c-Jun N-terminal kinase; NF-κB, nuclear factor kappa B; NQO1, NAD(P)H quinone dehydrogenase 1; Nrf2, nuclear factor erythroid 2-related factor 2; ROS, reactive oxygen species; TNF-α, tumor necrosis factor-alpha.

**Table 4:** Current Challenges and Future Directions for Translational Development of *Cirsium japonicum*.

Current Limitation	Impact	Future Direction	Key Ref.
Incomplete identification of active compounds	Difficult mechanistic interpretation	Advanced phytochemical profiling	[3,11]
Limited target validation	Weak causal evidence	CRISPR, target engagement studies	[3,10]
Insufficient pharmacokinetic data	Uncertain clinical translation	ADME and tissue distribution studies	[11]
Lack of standardized extracts	Poor reproducibility	Quality control using chemical markers	[6,11]
Few multi-omics studies	Limited systems-level understanding	Transcriptomics, proteomics, metabolomics integration	[10,69]
Limited clinical evidence	Poor translational applicability	Well-designed randomized clinical trials	[3]

## Data Availability

No new data were created or analyzed in this study. Data sharing is not applicable to this article.

## Abbreviations

AI	Artificial Intelligence
Akt	Protein Kinase B
AMPK	AMP-Activated Protein Kinase
ARE	Antioxidant Response Element
COX-2	Cyclooxygenase-2
ECM	Extracellular Matrix
ERK	Extracellular Signal-Regulated Kinase
GCLC	Glutamate-Cysteine Ligase Catalytic Subunit
GCLM	Glutamate-Cysteine Ligase Modifier Subunit
GPXs	Glutathione Peroxidases
HO-1	Heme Oxygenase-1
IKK	IκB Kinase
IL	Interleukin
iNOS	Inducible Nitric Oxide Synthase
JNK	c-Jun N-terminal Kinase
Keap1	Kelch-like ECH-Associated Protein 1
LC3-II	Microtubule-Associated Protein 1 Light Chain 3-II
MAPKs	Mitogen-Activated Protein Kinases
MASLD	Metabolic Dysfunction-Associated Steatotic Liver Disease
mTOR	Mechanistic Target of Rapamycin
NF-κB	Nuclear Factor-κB
NQO1	NAD(P)H Quinone Dehydrogenase 1
Nrf2	Nuclear Factor Erythroid 2-Related Factor 2
PI3K	Phosphoinositide 3-Kinase
ROS	Reactive Oxygen Species
SAR	Structure–Activity Relationship
SOD	Superoxide Dismutase
STAT3	Signal Transducer and Activator of Transcription 3
TGF-β	Transforming Growth Factor-β
TNF-α	Tumor Necrosis Factor-α
α-SMA	Alpha-Smooth Muscle Actin
